# Supplementary material for: Identification of miRNA–mRNA Regulatory Modules Involved in Lipid Metabolism and Seed Development in a Woody Oil Tree (Camellia oleifera)
Source: Cells. 2021 Dec 27;11(1):71. doi: 10.3390/cells11010071 (PMC8750442; doi:10.3390/cells11010071)
Supplement: Supplementary file 1 [file cells-11-00071-s001.zip › Figure S2.pdf]

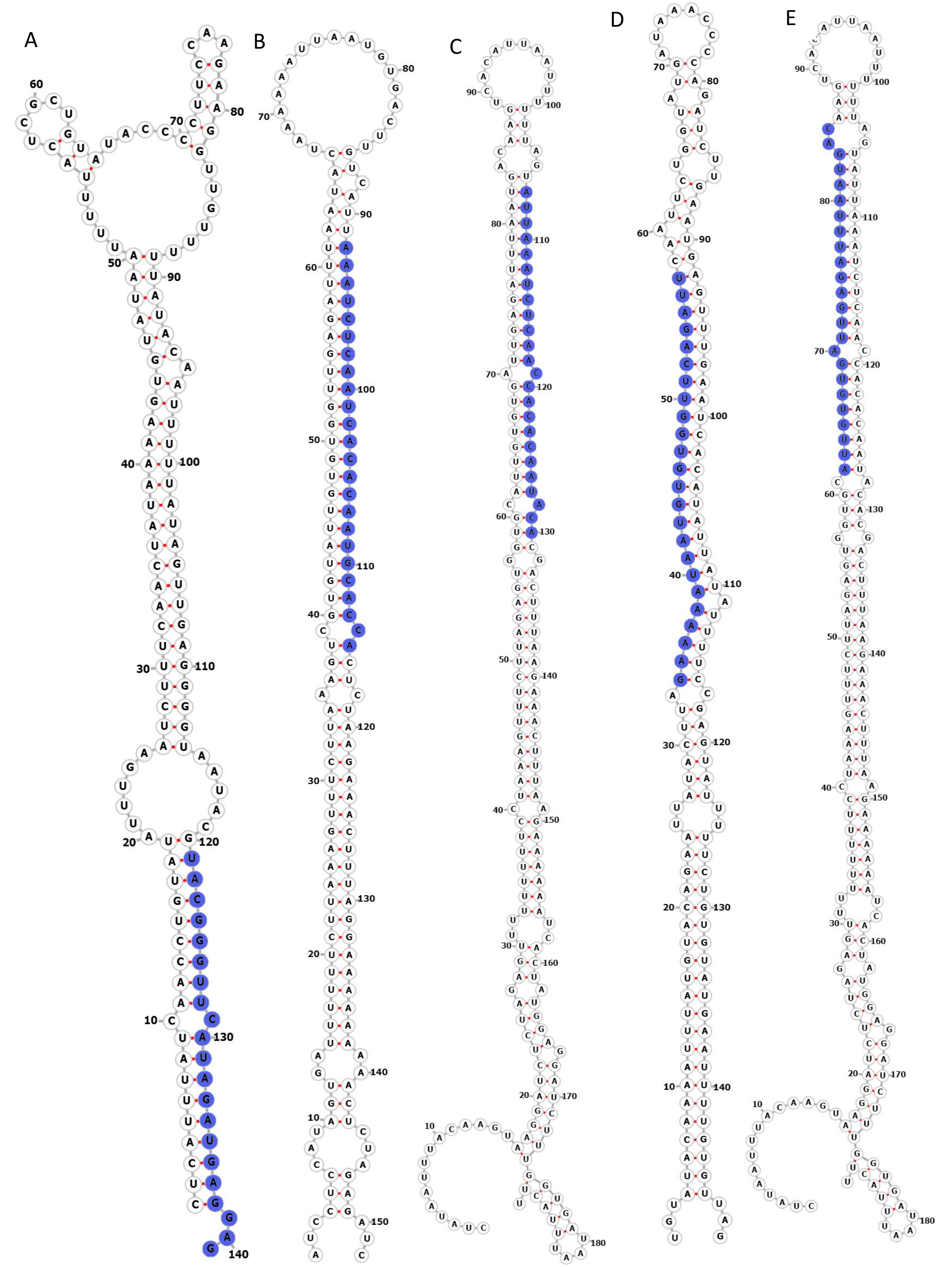

Figure S2 Secondary structures of the five most abundant novel miRNA precursors in tea oil camellia. A: col-miRn-5p-1103_1978; B：col-miRn-5p-2142_1228；C: col-miRn-3p-18781_243；D: col-miRn-3p-3803_812； E: col-miRn-3p-18137_250. Blue indicate segments corresponding to the mature miRNAs
