# Supplementary material for: Identification of miRNA–mRNA Regulatory Modules Involved in Lipid Metabolism and Seed Development in a Woody Oil Tree (Camellia oleifera)
Source: Cells. 2021 Dec 27;11(1):71. doi: 10.3390/cells11010071 (PMC8750442; doi:10.3390/cells11010071)
Supplement: Supplementary file 1 [file cells-11-00071-s001.zip › Table S10.pdf]

Table S10. Significantly differential expressed miRNAs in the same development stage seeds between two cultivars of 'M3' and 'M8' of tea oil camellia.

**Comparison stage**	**miRNA name**	***P*-value (*t*-test)**	**Up/Down**
M3T1 *vs*. M8T1	mtr-MIR2586a-p3_1ss15TC	2.85E-03	Up
	ath-MIR5645b-p5_2ss19TG21TC	1.15E-02	Up
	ath-MIR5645b-p3_2ss19TG21TC	1.15E-02	Up
	mtr-MIR2586a-p3_2ss4TG21CT	1.94E-02	Up
	hpe-miR166a_1ss5AC	3.35E-02	Up
M3T2 *vs*. M8T2	rco-miR156e_L+1R-1	8.38E-03	Down
	csi-miR1515_1ss16AG	1.41E-02	Up
	stu-miR8016_1ss7GT	1.63E-02	Up
	dpr-miR167a_R+1	1.86E-02	Down
	ccl-miR167a	2.53E-02	Down
	tcc-miR390a	3.52E-02	Down
	ath-miR390a-5p	3.52E-02	Down
	aly-miR167b-3p_1ss13AG	3.86E-02	Down
	ppe-miR172a-5p_1ss21GA	4.49E-02	Up
	han-miR156a_L+1	4.92E-02	Down
M3T3 *vs*. M8T3	col-miRn-3p-9080_426	1.36E-02	Down
	mtr-MIR2586a-p3_1ss15TC	1.49E-02	Down
M3T4 *vs*. M8T4	ghr-MIR7499-p3_2ss10GC17CG	1.24E-02	Down
	rco-miR167a_R+1	4.23E-02	Down
