# Supplementary material for: Identification of miRNA–mRNA Regulatory Modules Involved in Lipid Metabolism and Seed Development in a Woody Oil Tree (Camellia oleifera)
Source: Cells. 2021 Dec 27;11(1):71. doi: 10.3390/cells11010071 (PMC8750442; doi:10.3390/cells11010071)
Supplement: Supplementary file 1 [file cells-11-00071-s001.zip › Table S15.pdf]

Table S15 Function of target genes involved in seed traits in tea oil camellia in this study.

Target genes of identified miRNAs (gene annotation)	Function	References
**Lipid metabolism**		
ACC1 (Acetyl CoA Carboxylase 1)	The expressions of ACCase genes are significant to fatty acid and lipid biosynthesis	Wang, 2012
KAS1 (3-ketoacyl-ACP synthase I)	*KASI* is involved in the formation of C16 or C18 saturated fatty acids catalyzed by the fatty acid synthase complex	Yasuno *et al*., 2004
S-ACP-DES6 (Secretory acid phosphatase desaturase 6)	S-ACP DES is an archetypical member of a family of soluble fatty acid (FA) desaturases; one or more S-ACP-DES isozymes contribute to the 18:1 pool	Kachroo et al., 2007
KAS3B (3-ketoacyl-ACP synthase IIIB)	The 3-ketoacyl-*ACP* synthase II proteins are one of the most abundant enzymes in nature, as they are involved in the biosynthesis of fatty acids and natural products	Nofiani *et a*l., 2019
fabG (3-ketoacyl-acyl carrier protein reductase)	FabG is a member of the ketoacyl reductase family of proteins and is an essential enzyme for type II fatty acid biosynthesis	Lai and Cronan, 2004
Mcat ([acyl-carrier-protein] S-malonyltransferase)	[acyl-carrier-protein] S-malonyltransferase involved in lipid metabolism	Sang *et al*., 2013
FATB1 (fatty acyl-ACP thioesterase B)	*FATB* is a key enzyme gene that mainlycontrols the conversion of the 16-carbon palmitoyl-ACP to the palmitic acid.	Dörmann *et al*., 2000
MOD1 (enoyl-[acyl-carrier protein] reductase I)	It is a key enzyme in type II fatty-acid synthases that catalyzes the last step in each elongation cycle.	Heath *et al*., 2000
Δ9D (acyl-[acyl-carrier-protein] desaturase)	Increased *ACP-Δ9D* and *CoA-Δ9D* levels coupled with decreased *KASII* and *FAE1* activity is a critical event for high C16:1n7 accumulation in sea buckthorn berry	Ding *et al*., 2018
GAPN (glyceraldehyde-3-phosphate dehydrogenase)	Effects of overexpression of glyceraldehyde-3-phosphate dehydrogenase on growth and lipid accumulation in *Chlamydomonas reinhardtii*	Lee *et al*., 2013
**Seed size**		
ARF1 (auxin response factor 1)	MiR172 plays highly important roles in seed development by modulating ARF targeting in yellowhorn	Wang *et al*., 2021
ARF11 (auxin response factor 11)	ARFs are potentially involved in the seed size in seeds of sea buckthorn	Ding *et al*., 2019
ARF12 (auxin response factor 12)	MiR160 negatively regulates ARFs that significantly affect seed development in *A. thaliana*	Liu *et al*., 2010

ARF19 (auxin response factor 19)	Transcription factors of *SPL*, *MYB* and *ARF* involved in seed size/weight determination	Gupta *et al*., 2016<br>Khemka *et al*., 2021
ARF22 (auxin response factor 22)		
ARF6 (auxin response factor 6)		
CNR2 (cell number regulator 2)	CNR genes control plant fruit and organ size	Guo and Simmons, 2011
MED14 (Mediator of RNA polymerase II transcription subunit 14)	Mediator subunit OsMED14_1 plays an important role in rice development	Malik *et al*., 2020
MED18 (Mediator of RNA polymerase II transcription subunit 18)	MED18 interaction with distinct transcription factors regulates multiple plant functions	Lai *et al*., 2014
MED27 (Mediator of RNA polymerase II transcription subunit 27)	MED27 Variants Cause Developmental Delay, Dystonia, and Cerebellar Hypoplasia	**Meng *et al*., 2021**
MED28 (Mediator of RNA polymerase II transcription subunit 28)	MED28 Regulates Cell Growth through HMG Box-Containing Protein 1 (HBP1) in Human Breast Cancer Cells	Hsieh *et al*., 2013
MYB3 (Transcription factor MYB3)	Transcription factors of *SPL*, *MYB* and *ARF* involved in seed size/weight determination in chickpea	Khemka *et al*., 2021
MYB44 (Transcription factor MYB44)	The function of SlMYB as a candidate gene involved in fruit set process in tomato	Abdalla *et al*., 2013
MYB82 (Transcription factor MYB82)	Transcription factors of *SPL*, *MYB* and *ARF* involved in seed size/weight determination in chickpea	Khemka *et al*., 2021
**Growth and development**		
ACS1 (1-aminocyclopropane-1-carboxylate synthase)	Lc-ACS1 gene might be the key gene involved in fruit let abscission in litch	Wu *et al*., 2017
AFB2 (Protein AUXIN SIGNALING F-BOX 2)	The *AFB*2 gene is one of auxin receptor genes, which is involved in hormone regulation and signal transduction in plants	Schwab *et al*., 2005
COL13 (Zinc finger protein CONSTANS-LIKE 13)	AtCOL13 inhibits hypocotyl elongation of Arabidopsis under red light	Zhang, 2012
ERF115 (EREBP-like factor)	The ERF115–PAT1 complex plays an important role in full stem cell niche recovery	Heyman *et al*., 2016
SBP1 (Squamosa promoter-binding protein 1)	SBP1 is an important component of the atypical SCF complex, playing an unique role in the selfcompatibility system of gametes	Yu *et al*., 2019
SBP2 (Squamosa promoter-binding protein 2)	An SBP2-GFP fusion protein under the control of the GmSBP2 promoter accumulates in the vascular tissues of vegetative organs, which is consistent with the proposed involvement of SBP in sucrose transport-dependent physiological processes.	Freitas *et al*., 2007
SCL16 (Scarecrow-like protein 6)	SCL transcription factor family members in *Arabidopsis* involved in the growth and development of flowers, roots and stems	Sunkar et al., 2004

SPL4 (Squamosa promoter-binding-like protein 4)	Transcription factors of *SPL*, *MYB* and *ARF* involved in seed size/weight determination in chickpea	Khemka *et al*., 2021
T1R1 (transport inhibitor response 1)	The *T1R1* gene is one of auxin receptor genes, which is involved in hormone regulation and signal transduction in plants	Schwab *et al.,* 2005
TCP24 (Transcription factor TCP24)	*TCP*24 modulates secondary cell wall thickening and anther endothecium development	Han *et al*., 2015
**Resistance, yield and quality**		
*APUM*5 (Pumilio homolog 5)	APUM5 suppresses *Cucumber mosaic virus* infection via direct binding of viral RNAs	Huh *et al*., 2013
*BAG*7 (BAG family molecular chaperone regulator 7)	BAG protein family is an evolutionarily conserved kind of co-chaperone from yeast to animals and plants. The family members can perform several of cellular functions including stress response, growth, and programmed cell death	Wei *et al*. 2016
*BAM*1 (leucine-rich repeat receptor-like serine/threonine-protein kinase BAM1)	BAM1/2 receptor kinase signaling drives CLE peptide-mediated formative cell divisions in *Arabidopsis* roots	Crook *et al*., 2020
*CPR*30 (F-box protein CPR30)	The *CPR30* gene is a negative regulator of the defense response in *Arabidopsis*	Gou *et al*., 2010
*ERF*106 (EREBP-like factor)	This gene might have role in eliciting responses to combat stress and attribute the strong stress tolerant property associated with the plant	Tripathi *et al*., 2020
*MYB*4 (Myb-related protein Myb4)	*MYB4* gene is involved in drought stress response, and the regulation mechanism may be related to stomata	Ouyang, 2019
*NRT*1.7 (proton-dependent oligopeptide transporter, POT family)	The *Arabidopsis NLP7* gene regulates nitrate signaling via *NRT1.1*–dependent pathway in the presence of ammonium	Zhao *et al*., 2018
Os03g0214200 (Ninja-family protein Os03g0214200)	*Os03g0214200* is one of important candidate genes for *qLTGR3-1* of major QTL for germination ability of rice seeds at low temperature	Pan, 2018
*PAB*2 (20S proteasome subunit alpha 2)	Conserved expression of *Arabidopsis thaliana* poly (A) binding protein 2 (PAB2) in distinct vegetative and reproductive tissues	Palanivelu *et al*., 2010
*PYL*1 (Abscisic acid receptor PYL1)	This gene might be involved in the dormancy process of gladiolus corms regulated by ABA	Luo *et al*., 2019
*SGS*3 (Protein SUPPRESSOR OF GENE SILENCING 3)	BnSGS3 gene conferred moderate resistance to CMV; SGS3 and SGS2/SDE1/RDR6 are required for juvenile development and the production of trans-acting siRNAs in *Arabidopsis*	Peragine *et al*., 2004; Chen *et al*., 2015
*SPL*16 (Squamosa promoter-binding-like protein 16)	The *OsSPL16-GW7* regulatory module determines grain shape and simultaneously improves rice yield and grain quality	Wang *et al*., 2014
*XDH*1 (xanthine dehydrogenase/oxidas)	Antisense inhibition of xylitol dehydrogenase gene, *xdh1* from *Trichoderma reesei*	Wang *et al*., 2010

References:

Abdalla, A.H.S. Characterization of a myb gene from tomato (*solanum lycopersicum*) involved in fruit development. *Docencia*, 2013, http://hdl.handle.net/10251/44563

Chen, Q.; Wang, J.; Hou, M.S.; Liu, S.Y.; Cai, L. *Brassica napus* BnSGS3 gene sequence analysis,vector construction and resistance identification of transgenic *Arabidopsis thaliana* to viruses. *Chinese J. Oil Crop Sci.* 2015, 37(4) : 512-519

Crook, A.D.; Willoughby, A.C.; Hazak, O.; Okuda, S.; , Van Der Molen, K.R. ; Soyars, C.L.; Cattaneo, P.; Clark, N.M.; Sozzani, R.; Hothorn, M.; Hardtke, C.S.; Nimchuk, Z.L. BAM1/2 receptor kinase signaling drives CLE peptide-mediated formative cell divisions in *Arabidopsis* roots. *PNAS*, 2020, 117: 32750-32756

Ding, J.; Ruan, C.J.; Guan, Y.; Krishna, P. Identification of microRNAs involved in lipid biosynthesis and seed size in developing sea buckthorn seeds using high-throughput sequencing. *Sci. Rep.* 2018, 8: 4022

Ding, J.; Ruan, C.J.; Du, W.; Guan, Y. RNA-seq data reveals a coordinated regulation mechanism of multigenes involved in the high accumulation of palmitoleic acid and oil in sea buckthorn berry pulp. *BMC Plant Biol.* 2019, *19*, 207.

Dörmann, P.; Voelker, T.A.; Ohlrogge, J.B. Accumulation of palmitate in *Arabidopsis* mediated by the acyl-acyl carrier protein thioesterase *FATB1*. *Plant Physiol.* 2020, 123: 637-644

Freitas, R.L. , Carvalho, C.M. , Fietto, L.G., Loureiro, M.E. , Almeida, A.M., Fontes, E. Distinct repressing modules on the distal region of the sbp2 promoter contribute to its vascular tissue-specific expression in different vegetative organs. *Plant Mol. Biol.* 2007; 65(5): 603-614.

Gou, M.; Nan, S.; Zheng, J. ; Huai, J.; Wang, G. An f-box gene, cpr30, functions as a negative regulator of the defense response in arabidopsis. *Plant J.* 2010, 60(5): 757-770.

Guo, M.; Simmons, C.R. Cell number counts--the fw2.2 and CNR genes and implications for controlling plant fruit and organ size. *Plant Sci.* 2011; *181*, 1–7.

Gupta, M.; Bhaskar, P.B.; Sriram, S.; Sriram, S.; Wang, P.H. Integration of omics approaches to understand oil/protein content during seed development in oilseed crops. *Plant Cell Rep.* **2016**, *36*, 1–16.

Han, W.; Mao, Y.; Yang, J.; He, Y. *TCP*24 modulates secondary cell wall thickening and anther endothecium development. *Front. Plant Sci.* 2015, 6: 436.

Heath, R.J.; Su, N.; Murphy, C.K.; Rock, C.O. The Enoyl-[acyl-carrier-protein] reductases *FabI* and F*abL* from *Bacillus subtilis*. *J. Biol. Chem.* 2000, 275: 40128-40133

Heyman, J.; Cools, T.; Canher, B. The heterodimeric transcription factor complex ERF115–PAT1 grants regeneration competence. *Nat. Plants 2016,* 2, 16165

Hsieh, N.T. ; Huang, C.Y.; Chou, Y.H.; Ung, W.C.; Lee, M.F. Med28 regulates cell growth through hmg box-containing protein 1 (hbp1) in human breast cancer cells. *Faseb J.* 2013, 27:1028.1

Huh S.U; Kim,M.J.; Paek, K.H. *Arabidopsis* Pumilio protein APUM5 suppresses *Cucumber mosaic virus* infection via direct binding of viral RNAs. *PNAS*, 2013, 110: 779-784

Kachroo A.; Shanklin J.; Whittle E.; Lapchyk L.; Hildebrand D.; Kachroo P. The *Arabidopsis* stearoyl-acyl carrier protein-desaturase family and the contribution of leaf isoforms to oleic acid synthesis. *Plant Mol. Biol.* 2007, 63(2): 257-271

Khemka, N.; Rajkumar, M.S.; Garg, R.; Jain, M. Genome-wide profiling of miRNAs during seed development reveals their functional relevance in seed size/weight determination in chickpea. *Plant Direct* 2021, 5(3): e00299

Lai, C.Y.; Cronan, J.E. Isotation and characterization of β-ketoacyl-acyl carrier protein reductase (*fabG)* mutants of *Escherichia coli* and *Salmonella enterica* serovar Tyhimurium. *J. Bacteriol.* 2004, 186: 1869-1878

Lai, Z., Schluttenhofer, C., Bhide, K. Shreve J, Thimmapuram J, Lee SY, Yun DJ, Mengiste T. MED18 interaction with distinct transcription factors regulates multiple plant functions. *Nat Commun* 2014*,* 5, 3064

Lee, B.; Shin, S.E.; Choi, G.G.; Jeong, B.; Park, M.S.; Yang, J.W. Effects of overexpression of GAPDH on growth and lipid accumulation in *Chlamydomonas reinhardtii*. Asian Symposium on Innovative Biorefinary in Beijing, 2013.

Liu, P.P.; Montgomery, T.A.; Fahlgren, N.; Kasschau, K.D.; Carrington, J. C. Repression of AUXIN RESPONSE FACTOR10 by microRNA160 is critical for seed germination and post-germination stages. *Plant J.* 2010, *52*, 133–146.

Luo, X.; Yuan, Y.; Lu, H.; Wu, Y.; Yuan, L.; Jia, Y. Cloning and expression analysis of aba receptor gene pyl1 in gladiolus. *Mol. Plant Breeding,* 2019, 17: 3484-3489

Malik, N.; Ranjan, R.; Parida, S.K.; Agarwal, P.; Tyagi, A.K. Mediator subunit OsMED14_1 plays an important role in rice development. *Plant J.* 2020, 101(6):1411-1429.

Meng, L.Y.; Isohanni, P.; Shao, Y.R.; Graham, B.H.; Hickey, S.E.; Brooks, S.; Suomalainen, A.; Joset, P.; Steindl, K.; Rauch, A. MED27 variants cause developmental delay, dystonia, and cerebellar hypoplasia. *Ann. Neurol.* 2021, 89: 828-833

Nofiani, R.; Philmus, B.; Nindita, Y.; Mahmud, T. 3-ketoacyl-acp synthase (kas) iii homologues and their roles in natural product biosynthesis. *Medic. Chem. Commun. 2019,* 10:1517-1530

Ouyang, J. The mechanism of MYB4 gene involved in the response of drought stress in plants. Hefei University of Technology, A dissertation of Master's Degree, 2019

Palanivelu, R.; Belostotsky, D.A.; Meagher, R.B. Conserved expression of arabidopsis thaliana poly (a) binding protein 2 (pab2) in distinct vegetative and reproductive tissues. *Plant J.* 2010, 22(3), 199-210.

Pan, Z.Y. Mapping of QTLs controlling seed low temperature germinability and fine mapping and transcriptome analysis of qLTGR3-1 in rice. Huazhong Agricultural University, Dissertation of Master's Degree, 2018

Peragine, A.; Yoshikawa, M.; Wu, G.; Albrecht, H.L.; Poethig, R.S. SGS3 and SGS2/SDE1/RDR6 are required for juvenile development and the production of trans-acting siRNAs in *Arabidopsis*. *Genes Dev.* 2004, 18: 2368-79

Sang, Y.S.; Sang, H.K.; Velusamy, V.; Lee, Y.M. Comparative gene expression analysis in a highly anthocyanin pigmented mutant of colorless chrysanthemum. *Mol. Biol. Rep.* 2013, 40: 5177-5189.

Schwab, R.; Palatnik, J.F.; Riester, M.; Schommer, C.; Schmid, M.; Weigel, D. Specific effects of microRNAs on the plant transcriptome. *Dev. Cell* 2005, 8, 517–527.

Sunkar, R.; Zhu, J.K. Novel and stress-regulated microRNAs and other small RNAs from *Arabidopsis*. *Plant Cell* 2004; 16: 2001-2019

Tripathi, S.; Srivastava, Y. ; Sangwan, R.S. ; Sangwan, N.S. Mining and functional analysis of ap2/erf gene in. *Sci. Rep.* 2020, *10:* 4877.

Wang, B.M. Molecular cloning and functional study of ACCase genes from Camellia oleifera. Central Soouth University of Forestry and Technology, Ph.D dissertation, 2012.

Wang, L.; Ruan, C.J.; Bao, A.; Li, H. Small RNA proling for identification of microRNAs involved in regulation of seed development and lipid biosynthesis in yellowhorn. *BMC Plant Biol.* 2021, 21, 464.

Wang, S.; Li, S.; Liu, Q. The *OsSPL16-GW7* regulatory module determines grain shape and simultaneously improves rice yield and grain quality. *Nat Genet* 2014, 47**,** 949–954

Wang, T.H.; Zhong, Y.H.; Huang, W.; Liu, T.; You, Y.W. Antisense inhibition of xylitol dehydrogenase gene, xdh1 from trichoderma reesei. *Letters Appl. Microbiol.* 2010, *40*(6), 424-429.

Wei, R.M.; Xie, L.L.; Ouyang, X.; Zhang, Y.L.; Dai, Z.Z.; Liu, F. The Structure and Functional Characteristics of Plant BAG Protein Family. *Hunan Agri. Sci.* 2016, (09): 115-120 (in Chinese with an English abstract)

Wu, J.Y.; Li, C.Q.; Li, J.G.J. Isolation of acs1 gene and the relationship between its expression and fruitlet abscission in litchi. *J. Fruit Sci.* 2017, 34: 817-827

Yasuno, R.; von Wettstein-Knowles, P.; Wada, H. Identification and molecular characterization of the beta-Ketoacyl-[Acyl Carrier Protein] synthase component of the *Arabidopsis* mitochondrial fatty acid synthase. *J. Biol. Chem.* 2004, *279*, 8242–8251.

Yu, Z.; Ma, X.; Zeng, B.; Wang, J.; AbdkymAymt. Research progress on sbp1 gene of plant gametophytic self-incompatibility. *Mol. Plant Breeding* 2019, 17: 5285-5290 (in Chinese with an English abstract)

Zhang, Q. Analysis of signaling pathway in which AtCOL13 inhibits hypocotyl elongation of Arabidopsis under red light. South China Normal University, Dissertation for Master's Degree, 2012.

Zhao, L.; Zhang, W.; Yang, Y. The Arabidopsis NLP7 gene regulates nitrate signaling via NRT1.1–dependent pathway in the presence of ammonium. *Sci. Rep.* 8, 1487 (2018).
