# Supplementary figures and images for: Identification of miRNA–mRNA Regulatory Modules Involved in Lipid Metabolism and Seed Development in a Woody Oil Tree (Camellia oleifera)
Source: Cells. 2021 Dec 27;11(1):71. doi: 10.3390/cells11010071 (PMC8750442; doi:10.3390/cells11010071)

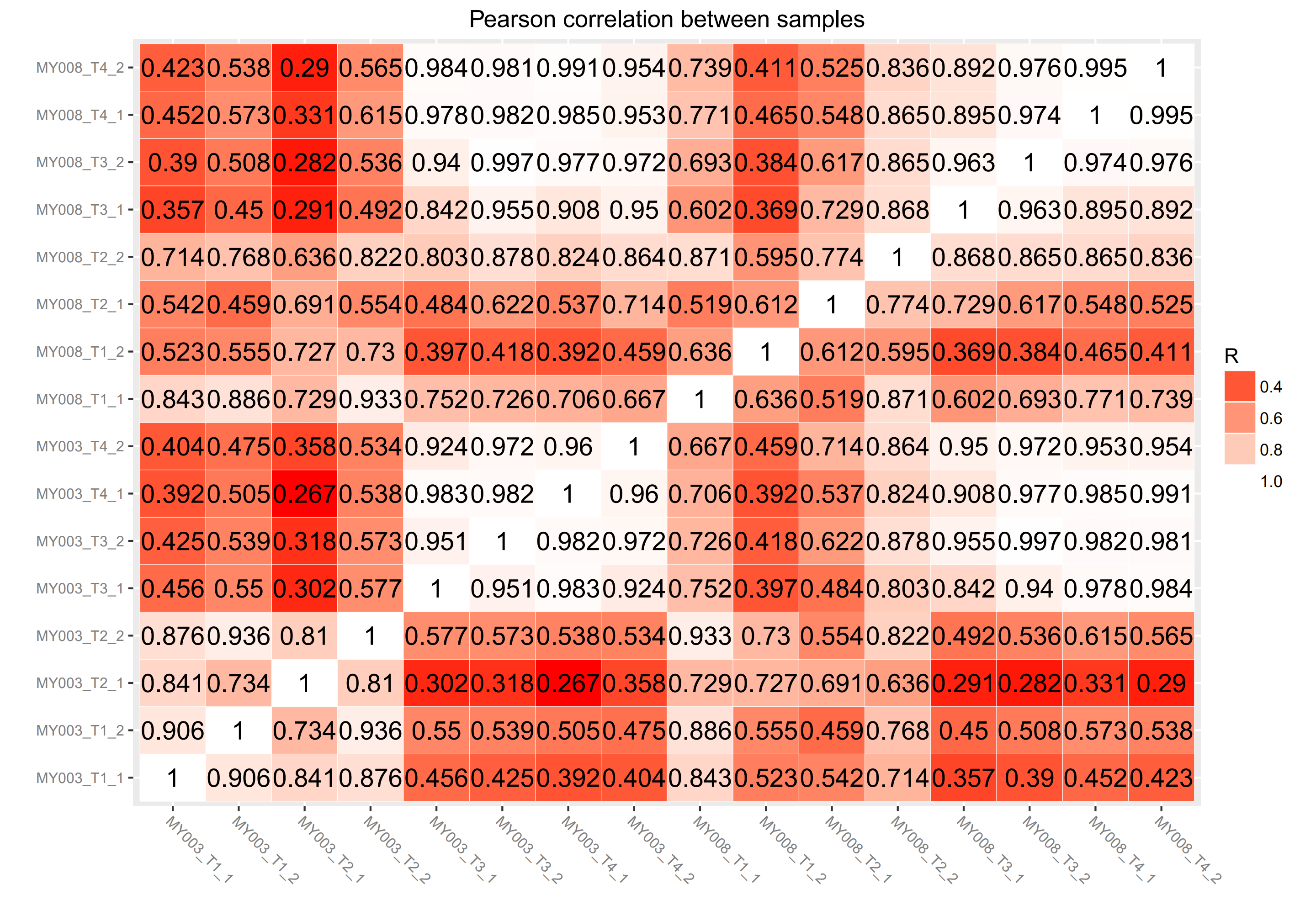

Supplement: Supplementary file 1 [file cells-11-00071-s001.zip › Figure S1.png]

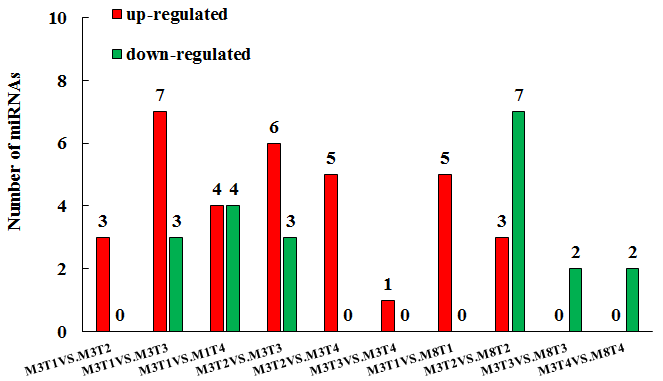

Supplement: Supplementary file 1 [file cells-11-00071-s001.zip › Figure S3.tif]
